# Relationship between Preoperative Echocardiographic Parameters and the Incidence of Postoperative Complications in Patients Undergoing Clipping of Unruptured Intracranial Aneurysms: A Retrospective Cohort Study

**DOI:** 10.3390/medicina59101697

**Published:** 2023-09-22

**Authors:** Yong-Seok Park, Seung-Ah Lee, Ji-Hoon Sim, Baehun Moon, Kyoung-Sun Kim, Seungil Ha, Jung-Hoon Choi, Sung-Hoon Kim

**Affiliations:** 1 Department of Anesthesiology and Pain Medicine, Asan Medical Center, University of Ulsan College of Medicine, Seoul 05505, Republic of Korea; yongparkanesth@gmail.com (Y.-S.P.); y2kaft@gmail.com (J.-H.S.);; 2 Department of Cardiology, Asan Medical Center, University of Ulsan College of Medicine, Seoul 05505, Republic of Korea; salee0602@gmail.com

**Keywords:** unruptured intracranial aneurysm, preoperative echocardiography, postoperative complication

## Abstract

*Background and Objectives*: Preoperative echocardiography is widely performed in patients undergoing major surgeries to evaluate cardiac functions and detect structural abnormalities. However, studies on the clinical usefulness of preoperative echocardiography in patients undergoing cerebral aneurysm clipping are limited. Therefore, this study aimed to investigate the correlation between preoperative echocardiographic parameters and the incidence of postoperative complications in patients undergoing clipping of unruptured intracranial aneurysms. *Materials and Methods*: Electronic medical records of patients who underwent clipping of an unruptured intracranial aneurysm from September 2018 to April 2020 were retrospectively reviewed. Data on baseline characteristics, laboratory variables, echocardiographic parameters, postoperative complications, and hospital stays were obtained. Univariable and multivariable logistic regression analyses were performed to identify independent variables related to the occurrence of postoperative complications and prolonged hospital stay (≥8 d). *Results*: Among 531 patients included in the final analysis, 27 (5.1%) had postoperative complications. In multivariable logistic regression, the total amount of crystalloids infused (1.002 (1.001–1.003), *p* = 0.001) and E/e’ ratio (1.17 (1.01–1.35), *p* = 0.031) were significant independent factors associated with the occurrence of a postoperative complication. Additionally, the maximal diameter of a cerebral aneurysm (1.13 (1.02–1.25), *p* = 0.024), total amount of crystalloids infused (1.001 (1.000–1.002), *p* = 0.031), E/A ratio (0.22 (0.05–0.95), *p* = 0.042), and E/e’ ratio (1.16 (1.04–1.31), *p* = 0.011) were independent factors related to prolonged hospitalization. *Conclusions*: Echocardiographic parameters related to diastolic function might be associated with postoperative complications in patients undergoing clipping of unruptured intracranial aneurysms.

## 1. Introduction

Preoperative echocardiography is widely performed in patients undergoing major surgeries to evaluate cardiac functions and detect structural abnormalities. Echocardiographic parameters related to systolic and diastolic functions are known to be associated with postoperative outcomes, especially in cardiac surgery [1,2,3]. Regional wall motion index and tissue Doppler indexes are useful tools to evaluate preoperative cardiac function and predict postoperative recovery [4,5]. However, in non-cardiac surgeries, there are controversies about the predictive ability of preoperative echocardiography for postoperative outcomes [6,7]. There are reports that preoperative echocardiography is an independent factor in predicting the risk of postoperative cardiac complications in major non-cardiac surgery [6], whereas another study reports routine preoperative echocardiography does not have a relationship with either in-hospital mortality or a decrease in postoperative complications in hip fracture surgeries [7]. Therefore, the prognostic value of preoperative echocardiography varies according to each surgical patient group.

Unruptured intracranial aneurysms have a prevalence rate of 3.2% and pose a potentially serious risk to the patient, as ruptured intracranial aneurysms cause subarachnoid hemorrhage, leading to a high mortality rate [8,9,10]. Surgical clipping, a conventional treatment for intracranial aneurysms, has been performed in various centers and is known to have superior outcomes compared to natural disease progress [8,11,12,13]. Although simple coil embolization demonstrates lower short-term mortality, it presents higher retreatment rates compared to surgical clipping, while both methods exhibit similar long-term mortality [14]. Therefore, the choice of surgical/non-surgical treatment for aneurysm clippings should be based on variations in coiling techniques and factors like aneurysm size and location.

The relationship between echocardiographic LV dysfunction findings and prognosis in patients with aneurysmal subarachnoid hemorrhage has been reported. Changes in echocardiographic findings, including regional wall motion abnormalities, are commonly observed in patients with aneurysmal subarachnoid hemorrhage [15], and they can be improved after treatment with clipping [16]. Moreover, in patients with subarachnoid hemorrhage, echocardiographic findings of regional wall motion abnormality and neurogenic cardiomyopathy have been shown to provide predictive information of in-hospital mortality [17]. However, studies on preoperative echocardiographic abnormalities in patients undergoing clipping of unruptured aneurysms are limited.

In this study, we aimed to investigate the correlation between preoperative echocardiographic parameters and postoperative outcomes, including the incidence of postoperative complications and prolonged hospitalization in patients undergoing clipping of unruptured intracranial aneurysms.

## 2. Materials and Methods

### 2.1. Ethics Approval and Consent to Participate

This study was approved by the institutional review board of Asan Medical Center (No. 2021-0928). The requirement for written informed consent was waived owing to the retrospective nature of the study.

### 2.2. Study Population

Electronic medical records of patients who underwent clipping of unruptured intracranial aneurysm from September 2018 to April 2020 were retrospectively reviewed. Patients aged below 18 years who underwent emergency or combined procedures were excluded. Patients without preoperative echocardiography were also excluded.

### 2.3. Data Collection

All data, including baseline characteristics, laboratory variables, echocardiographic parameters, intraoperative variables, and outcome variables, were collected from an electronic medical recording system (Asan Medical Information System 3.0, South Korea). Age, sex, body mass index, underlying disease, and location and maximal diameter of intracranial aneurysm were included in baseline characteristics. Intraoperative variables included transfusion, administration of albumin, mannitol infusion, total infused volume of crystalloids, and urine output. Preoperative echocardiographic variables included valvular abnormality; left ventricular (LV) internal diameter; left atrium dimension; LV posterior wall thickness; LV end-diastolic and end-systolic volume; interventricular septal thickness; LV ejection fraction; LV mass; peak E and A wave velocity; E/A ratio; deceleration time; S, E, and A velocity on tissue Doppler; E/e’ ratio; peak systolic tricuspid pressure gradient; and wall motion index.

### 2.4. Anesthesia Management

Anesthetic management followed routine anesthesia protocol for intracranial aneurysm clipping of our center. Approximately 25–50 mg of pethidine was administered intravenously after applying non-invasive blood pressure measurement, pulse oximetry, and routine monitors. Then, an arterial catheter was placed for continuous blood pressure monitoring after lidocaine was locally infiltrated. For anesthesia induction, 2 mg/kg of intravenous propofol and 0.8–1 mg/kg of intravenous rocuronium was injected. Remifentanil and propofol were injected via a target-controlled injection pump (Orchestra, Fresenius Vial, Brezins, France). To prevent a sudden increase in blood pressure, intubation was performed gently with the administration of 10–30 mg intravenous esmolol as required. After intubation, ventilation was calibrated to an arterial carbon dioxide pressure of 35 ± 2 mmHg, and the patient was ventilated with an air/oxygen mixture (fraction of inspired oxygen, 0.5). To analyze the appropriate depth of anesthesia, the bispectral index (Aspect Medical Systems Inc., Framingham, MA, USA) was used (target index 40 ± 5). When hypotension or bradycardia occurred, a continuous infusion of phenylephrine (0.5–3.0 mg/h) and/or bolus injection of atropine (0.25–0.5 mg) was administered to maintain the systolic blood pressure within 100–150 mmHg and to increase the heart rate over 45 beats per minute.

### 2.5. Evaluation of the Outcomes

The primary outcome was overall postoperative complications, which included neurological and non-neurological complications. Neurological complications included postoperative motor weakness, altered mentality, dysarthria, dysphagia, hydrocephalus, brain edema, cerebral infarction, and brain death. Non-neurological complications included postoperative cardiac events, surgical site hemorrhage, pneumonia, pulmonary edema, elevated liver enzymes, and other complications of non-neurological causes. The secondary outcome was prolonged hospital stay defined as hospitalization of ≥8 d.

### 2.6. Statistical Analysis

Baseline characteristics, perioperative variables, and preoperative echocardiographic variables are expressed as mean (standard deviation), median (interquartile range), or n (proportion). Univariable and multivariable logistic regression analyses were performed to identify the independent variables associated with the occurrence of postoperative complication and prolonged hospital stay. The stepwise selection approach was used to select variables for multivariable logistic regression. The model with the lowest Akaike information criterion was selected as the final model. The odds ratio, 95% confidence interval, and *p*-value were illustrated within the univariable and multivariable analyses. All statistical tests were performed using R 4.1.0.

## 3. Results

**Figure 1:**
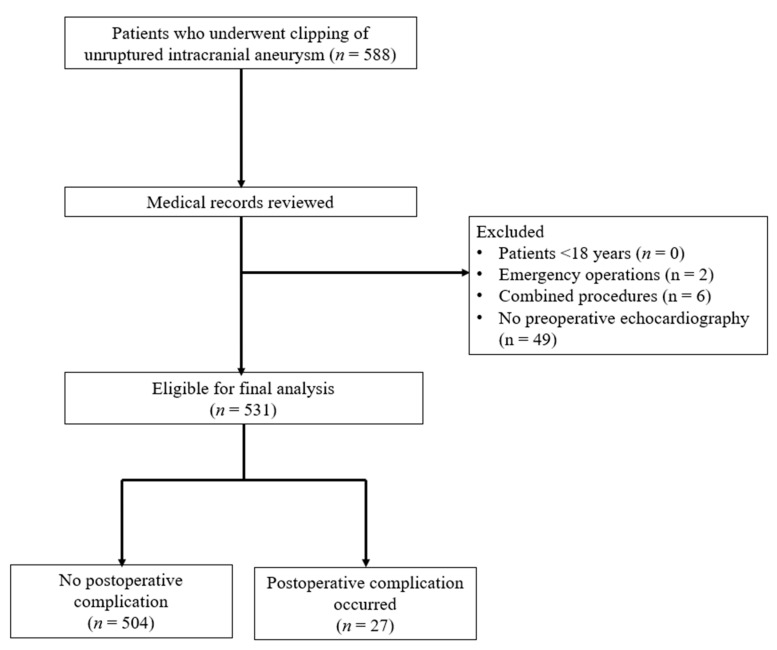
Study flowchart.

Electronic medical records of 588 patients who underwent clipping of unruptured intracranial aneurysm were reviewed. Among the 588 patients, 2 who underwent emergency operations and 6 who received combined procedures were excluded. Forty-nine patients without preoperative echocardiography records were also excluded. Finally, 531 patients (369 female, 61.6 ± 8.2 years) were included in the final analysis (Figure 1). Demographic statistics and preoperative echocardiographic parameters are shown in Table 1 and Table 2, respectively. Of all patients included in the final analysis, 60.8% had hypertension, with a mean BMI of 25.2 kg/m^2^. The location of the aneurysm was most common in the middle cerebral artery at 41.6%, followed by the anterior cerebral artery at 20.5%. The maximal diameter of the aneurysm was 5.0 ± 2.3 mm (Table 1). Overall, valvular abnormal findings in echocardiography were detected in 17 patients (3.2%), with no significant difference in incidence between the non-complication and complication groups (3.2% vs. 3.7%; *p* = 0.594). Quantitative variables such as LV internal diameter, LV wall thickness, end-systolic volume, end-diastolic volume, LV ejection fraction, LV mass, peak E wave and A wave velocity, deceleration time, and tissue Doppler parameters also did not show significant differences between the groups with and without postoperative complications (Table 2). Among the echocardiographic parameters, the E/e’ ratio was the only variable that showed a significant difference between those who developed postoperative complications and those who did not (10.28 ± 2.87 vs. 8.97 ± 2.57; *p* = 0.008) (Table 2).

Among the 531 patients, 27 (5.1%) had postoperative complications. Neurologic complications were the most frequent (55.6%), followed by wound problems and respiratory complications (Table 3). Among the 15 neurological complications, cerebral infarction was the most frequent with 9 cases, followed by intracranial hemorrhage and third nerve palsy. Among respiratory complications, pulmonary edema was the most common. There was only one case of cardiac postoperative complication in which new-onset atrial fibrillation occurred.

In multivariable logistic regression analysis, the total amount of crystalloids infused (adjusted odds ratio 1.002 [95% confidence interval 1.001–1.003], *p* = 0.001) and E/e’ ratio (1.17 [1.01–1.35], *p* = 0.031) were independent variables associated with postoperative complications (Table 4). The mean E/e’ ratio of patients with postoperative complications was higher than that in patients without postoperative complications (8.97 ± 2.57 vs. 10.28 ± 2.87, *p* = 0.008) (Table 2). The variables that were statistically significant in the univariable analysis (maximum diameter of the cerebral aneurysm, RBC transfusion, and urine output) did not remain as significant independent variables associated with postoperative overall complications in the multivariable analysis (Table 4). The secondary outcome, prolonged hospitalization, was independently associated with the maximum diameter of a cerebral aneurysm (1.13 (1.02–1.25), *p* = 0.024), total amount of crystalloids infused (1.001 (1.000–1.002), *p* = 0.031), E/A ratio (0.22 (0.05–0.95), *p* = 0.042), and E/e’ ratio (1.16 (1.04–1.31), *p* = 0.011) (Table 5). RBC transfusions and urine output, variables that were significantly associated with prolonged hospital stay in the univariable analysis, did not remain as significant independent variables in the multivariable analysis.

## 4. Discussion

In the present study, diastolic-function-related echocardiographic parameters, such as E/A and E/e’ ratios, showed a relationship with postoperative outcomes. The E/e’ ratio was associated with both postoperative overall complications and prolonged hospitalization. Other than echocardiographic parameters, the total amount of infused crystalloids was an independent factor associated with postoperative overall complications, while the maximum diameter of a cerebral aneurysm and the total infused crystalloids were associated with prolonged hospitalization.

Doppler echocardiography is widely used as a modality to evaluate diastolic function [18]. The E/e’ ratio is a ratio of the mitral inflow’s early peak flow to the mitral annulus’ early velocity and is used as an excellent parameter representing diastolic filling tissue Doppler imaging index [19]. In clinical practice, E/e’ < 8 suggests normal LV filling pressure, and E/e’ > 15 indicates high LV filling pressure [19,20]. The E/e’ ratio is known to be related to the prognosis of patients undergoing surgery or with cardiovascular disease. In patients who underwent non-cardiac surgery, preoperative echocardiography E/e’ > 15 predicted postoperative pulmonary edema and major adverse events [20]. There are also some studies which showed an association between high E/e’ and decreased survival rate in patients with acute coronary syndrome or severe aortic stenosis [21,22,23]. In our study, the E/e’ ratio had a strong association with the incidence of postoperative complication and prolonged hospitalization in patients undergoing clipping of unruptured intracranial aneurysm. Considering that a high E/e’ ratio suggests diastolic dysfunction, our data revealed a possibility of a correlation between diastolic function and postoperative outcome among these patients and seems consistent with previous studies that revealed the consequence of a high E/e’ ratio. However, as neurologic complications accounted for more than half of the overall complications and cardiac events were rare in our data, the mechanism explaining the relationship between ventricular diastolic function and postoperative outcome in these patients might be different from that reported in other studies.

Several studies exist on the association between cardiac diastolic function and neurologic outcomes. In a multicenter study evaluating the association of echocardiographic parameters related to LV diastolic function with ischemic stroke in patients with atrial fibrillation, the E/e’ ratio was significantly greater in those with a stroke history, and E/e’ ratio was also a significant independent predictor of ischemic stroke in multivariable analysis [24]. Although this correlation is partially explained by worsening atrial function and blood stasis due to elevated LV filling pressures in patients with atrial fibrillation [24], the study did not establish a definite causal relationship or a clear mechanism connecting LV diastolic dysfunction and ischemic stroke, especially due to the limitations of a retrospective study. In a study reporting the association of central retinal artery occlusion with vascular endothelial injury and LV diastolic dysfunction, impaired LV diastolic function was associated with lower flow-mediated dilatation of the brachial artery and thicker intima-media complex thickness, suggesting reciprocal remodeling between cardiac structures and the arterial wall [25]. Given that the evidence points to a significant involvement of inflammation in both myocardial fibrosis and diastolic dysfunction [26], the findings of these studies may indirectly explain the mechanisms of diastolic dysfunction and neurovascular outcomes. In other words, it can be assumed that the diastolic function of the heart and changes in vascular status may affect each other or have a common mechanism. Other studies reporting the relationship between diastolic dysfunction and the occurrence of cerebral infarction have similarly described vascular endothelial cell damage and hypercoagulability due to sympathetic nervous system hyperactivity as the mechanism of the relationship [27,28]. Meanwhile, Sacre et al. were the first to report an association between mild cognitive impairment and diastolic dysfunction [29]. In their study, individuals who exhibited evidence of LV diastolic dysfunction through echocardiographic markers, including E/e’ ratio, showed about twice the likelihood of experiencing concurrent mild cognitive impairment, compared to those with normal cardiac function. This association persisted even after adjusting for factors including age, gender, and other clinical variables. The authors suggest that possible connections between diastolic dysfunction and mild cognitive impairment involve potential coinciding factors such as increased arterial stiffness and reduced cardiac functional reserve, both of which can contribute to impaired cerebral circulation. Another study investigating the association between diastolic dysfunction and subarachnoid hemorrhage suggested a multifactorial mechanism, such as hypertension and old age but still only partially explained the relationship between diastolic dysfunction and cerebrovascular complications [30]. Overall, although there are several studies linking cardiac diastolic function to neurological outcomes, the mechanisms are not clearly established and have been partially explained by arterial stiffness, endothelial cell damage, and hypercoagulability due to elevated LV filling pressure, atrial dysfunction, and sympathetic hyperactivity.

The present study is in line with the aforementioned studies in that it investigated the association between cardiac diastolic dysfunction and neurologic outcome. However, the study differs from previous studies in that study subjects were specifically limited to patients undergoing clipping of intracranial aneurysm via craniotomy. To our knowledge, this is the first study to examine the association between cardiac diastolic function and postoperative outcome in patients undergoing aneurysm clipping. While establishing a clear mechanism for the results of this study is difficult, we believe that endothelial injury, arterial stiffness, and hypercoagulability may explain some of the findings. These explanations are consistent with previous studies on diastolic dysfunction and neurological outcomes [24,27,28,29,30,31]. Noxious stimulation and alterations in autonomic nervous system during general anesthesia and craniotomy, as well as direct manipulation of cerebral blood vessels, are thought to have additive effects on vulnerability due to diastolic dysfunction. Despite advances in surgical techniques, there are still instances of morbidity associated with this procedure. One significant and undesirable complication is the occlusion of the parent artery or small hidden perforators by clipping, leading to permanent neurological deficits [32]. This is why intraoperative somatosensory evoked potential and motor evoked potential monitoring are routinely performed in aneurysm clipping surgery in many centers, although the diagnostic test accuracy has been reported differently in different studies [33]. The possibility of postoperative neurologic complication by perforator injury also may be an example of how preservation of blood flow to the branches and perforator during surgical manipulation can have a significant impact on postoperative morbidity. In other words, it can be assumed that in patients with diastolic dysfunction, changes in arterial stiffness and endothelial status are likely to reduce the ability to compensate for changes in blood flow to the perforators and branches caused by surgical manipulation. Considering that this study involved patients with unruptured aneurysms undergoing elective surgery, which generally carries a lower incidence of postoperative complications compared to cases with ruptured aneurysms or emergency cases [34], it is noteworthy that the E/e’ ratio continues to stand out as a significant independent variable for postoperative complications even in the population at relatively lower risk.

E/A ratio, a ratio of early diastolic peak flow to late diastolic peak flow, has also been used as a marker of diastolic function [35]. E/A ratio > 2.5 is known to be associated with high left atrial pressure in patients with systolic dysfunction, although it is less sensitive than the deceleration time [18]. However, as the diastolic dysfunction progresses, there is a stage in which the E/A ratio returns to the normal range (pseudonormalization in stage 2 diastolic dysfunction) [36]; thus, it is difficult to use the E/A ratio as a linear index for the degree of diastolic dysfunction. In this study, the E/A ratio was an index independently associated only with the secondary outcome and prolonged hospital stay; however, it did not show a significant relationship with the primary outcome. Furthermore, the odds ratio of the E/A ratio for prolonged hospital stay was 0.22 [0.05–0.95], which indicates that the higher the E/A ratio, the smaller the odds of the secondary outcome. Therefore, rather than using the E/A ratio alone as an indicator of diastolic dysfunction, it seems that indicators, such as the E/e’ ratio, should also be considered.

There are several limitations in the current study. First, as a retrospective study, unexpected biases may have affected the results and interpretation of the study. As data were obtained by reviewing electronic medical records, variables related to outcome could be omitted from the data acquisition process or the medical record itself. Owing to the limitations of this retrospective study, we are planning a prospective study on this topic in future. Second, the incidence of cardiovascular complications related to the echocardiographic parameters was too low to perform subgroup analysis. Although cardiovascular complications can be a direct indicator of the impact of diastolic dysfunction on postoperative prognosis, our study population had few cardiovascular comorbidities and stable perioperative vital signs, resulting in only one cardiovascular complication. Considering that most of the complications in this study were neurological complications, such as cerebral infarction, there seems to be a need for research on the association between diastolic dysfunction and cerebrovascular status.

## 5. Conclusions

There is a possibility of an association between preoperative echocardiography parameters, which reflect diastolic function, and postoperative outcomes, including the incidence of postoperative complication and hospital stay, in patients undergoing clipping of unruptured intracranial aneurysms. Therefore, a prospective study to identify the value of preoperative echocardiographic parameters in predicting postoperative outcomes in this population is warranted.

## Figures and Tables

**Table 1:** Baseline characteristics and perioperative variables.

Variables	Overall (N = 531)
**Baseline characteristics**	
Age (year)	61.6 (8.2)
Sex: male	162 (30.5%)
Body mass index (kg/m^2^)	25.2 (3.1)
Diabetes mellitus	67 (12.6%)
Hypertension	323 (60.8%)
Location of aneurysm	
Anterior cerebral artery	109 (20.5%)
Middle cerebral artery	221 (41.6%)
Internal carotid artery	78 (14.7%)
Multiple locations	118 (22.2%)
Other locations	5 (0.9%)
Maximal diameter (mm)	5.0 (2.3)
**Preoperative laboratory variables**	
Hemoglobin (g/dL)	13.0 (1.3)
Platelet (10^3^/μL)	237 (57)
Prothrombin time (INR)	0.98 (0.06)
Creatinine (mg/dL)	0.8 (0.4)
Sodium (mmol/L)	141 (2)
Potassium (mmol/L)	4.3 (0.3)
Albumin (g/dL)	3.9 (0.3)
**Intraoperative variables**	
Total crystalloids infused (mL)	1476 (458)
5% albumin infusion	4 (0.8%)
RBC transfusion	88 (16.6%)
Mannitol infusion	11 (2.1%)
Urine output (mL)	696 (482)

Values are expressed as mean (SD) or n (proportion). RBC, red blood cell; INR, international normalized ratio; and SD, standard deviation.

**Table 2:** Preoperative echocardiographic variables.

Variables	No Complication (N = 504)	Complication (N = 27)	Overall (N = 531)	*p*-Value
Valvular abnormality	16 (3.2%)	1 (3.7%)	17 (3.2%)	0.594
LV internal diameter, systole (mm)	28.0 (4.5)	28.2 (4.4)	28.0 (4.5)	0.919
LV internal diameter, diastole (mm)	46.8 (4.6)	46.6 (5.3)	46.8 (4.6)	0.893
Left atrium dimension (mm)	35.6 (5.0)	36.6 (5.2)	35.7 (5.0)	0.264
LV posterior wall thickness, systole (mm)	13.9 (1.8)	13.9 (2.5)	13.9 (1.8)	0.986
LV posterior wall thickness, diastole (mm)	8.8 (1.1)	8.7 (1.3)	8.8 (1.1)	0.746
End-systolic volume (mL)	30.7 (10.3)	28.9 (10.5)	30.6 (10.3)	0.408
End-diastolic volume (mL)	84.5 (22.1)	80.2 (20.8)	84.3 (22.0)	0.414
Interventricular septal thickness, systole (mm)	13.3 (1.9)	12.9 (1.7)	13.3 (1.9)	0.295
Interventricular septal thickness, diastole (mm)	8.9 (1.2)	8.7 (0.9)	8.9 (1.2)	0.287
LV ejection fraction (%)	64.0 (4.3)	64.4 (5.4)	64.0 (4.3)	0.690
LV mass (g)	141 (34)	137 (5)	141 (34)	0.327
Peak E wave velocity (cm/s)	65.1 (15.6)	68.4 (20.0)	65.3 (15.9)	0.404
Peak A wave velocity (cm/s)	73.8 (15.1)	77.2 (12.6)	73.9 (15.0)	0.235
E/A ratio	0.91 (0.27)	0.86 (0.21)	0.90 (0.27)	0.343
Deceleration time (ms)	217 (45)	217 (64)	217 (46)	0.967
TDI S velocity (cm/s)	7.7 (1.4)	7.4 (1.3)	7.7 (1.4)	0.285
TDI E velocity (cm/s)	6.7 (3.4)	6.2 (1.7)	6.6 (3.3)	0.227
TDI A velocity (cm/s)	10.1 (6.3)	9.7 (1.6)	10.1 (6.2)	0.777
E/e’ ratio	8.97 (2.57)	10.28 (2.87)	9.04 (2.59)	0.008
Wall motion index	1.01 (0.09)	1.04 (0.19)	1.01 (0.09)	0.129

Values are expressed as the mean (SD) or n (proportion). LV, left ventricle; TDI, tissue Doppler imaging; and SD, standard deviation.

**Table 3:** Types of postoperative complications.

Complication	Number (%)
**Neurologic**	**15 (55.6%)**
Cerebral infarction	9 (33.3%)
Third cranial nerve palsy	2 (7.4%)
Intracranial hemorrhage	4 (14.8%)
**Respiratory**	**3 (11.1%)**
Pulmonary edema	2 (7.4%)
Desaturation	1 (3.7%)
**Cardiac**	**1 (3.7%)**
Atrial fibrillation	1 (3.7%)
**Other**	**8 (29.6%)**
Wound problem	4 (14.8%)
Fever	3 (11.1%)
Anaphylactic shock	1 (3.7%)

**Table 4:** Univariable and multivariable regression analyses for overall postoperative complications.

	Univariable Analysis		Multivariable Analysis	
Variables	Odds Ratio (95% CI)	*p*-Value	Odds Ratio (95% CI)	*p*-Value
**Baseline characteristics**				
Age (year)	1.05 (1.01–1.09)	0.053	1.02 (0.96–1.08)	0.620
Sex: male	0.87 (0.45–1.79)	0.744		
Body mass index (kg/m^2^)	0.93 (0.83–1.03)	0.254		
Diabetes mellitus	1.62 (0.64–3.58)	0.347		
Hypertension	0.68 (0.35–1.31)	0.329		
**Location of aneurysm**				
Anterior cerebral artery	reference			
Middle cerebral artery	0.68 (0.26–1.90)	0.519		
Internal carotid artery	2.05 (0.76–5.79)	0.236		
Multiple locations	1.31 (0.49–3.68)	0.652		
Other locations	5.20 (0.45–32.08)	0.172		
Maximal diameter (mm)	1.19 (1.06–1.32)	0.01	1.14 (0.98–1.32)	0.091
**Preoperative laboratory variables**				
Hemoglobin (g/dL)	0.80 (0.62–1.03)	0.152		
Creatinine (mg/dL)	1.10 (0.37–1.74)	0.812		
Albumin (g/dL)	0.40 (0.13–1.21)	0.172		
**Intraoperative variables**				
Total crystalloids infused (mL)	1.002 (1.001–1.002)	<0.001	1.002 (1.001–1.003)	0.001
RBC transfusion	4.51 (2.28–8.78)	<0.001	2.09 (0.81–5.38)	0.128
Urine output (mL)	1.001 (1.000–1.001)	0.014	0.999 (0.998–1.000)	0.260
**Echocardiographic parameters**				
Valvular disease	1.17 (0.12–4.87)	0.879		
End-diastolic volume (mL)	0.99 (0.97–1.01)	0.326		
LV ejection fraction (%)	1.03 (0.95–1.11)	0.617		
LV mass (g)	1.00 (0.99–1.01)	0.570		
E/A ratio	0.51 (0.11–1.88)	0.443		
Deceleration time (ms)	1.00 (0.99–1.01)	0.955		
TDI S velocity (cm/s)	0.84 (0.64–1.08)	0.262		
TDI E velocity (cm/s)	0.89 (0.70–1.04)	0.367		
TDI A velocity (cm/s)	0.98 (0.83–1.03)	0.743		
E/e’ ratio	1.17 (1.05–1.30)	0.015	1.17 (1.01–1.35)	0.031
Wall motion index	4.60 (0.49–23.33)	0.149		

CI, confidence interval; RBC, red blood cell; LV, left ventricle; and TDI, tissue Doppler imaging.

**Table 5:** Univariable and multivariable regression analyses for prolonged hospital stays.

	Univariable Analysis		Multivariable Analysis	
Variables	Odds Ratio (95% CI)	*p*-Value	Odds Ratio (95% CI)	*p*-Value
**Baseline characteristics**				
Age (year)	1.02 (0.99–1.05)	0.270		
Body mass index (kg/m^2^)	0.95 (0.88–1.02)	0.260		
Hypertension	0.65 (0.42–1.00)	0.099	0.56 (0.31–1.02)	0.056
Location of aneurysm				
Anterior cerebral artery	Reference			
Middle cerebral artery	0.78 (0.42–1.46)	0.496		
Internal carotid artery	1.21 (0.58–2.50)	0.661		
Multiple locations	1.33 (0.70–2.56)	0.467		
Other locations	11.08 (2.31–61.70)	0.012		
Maximal diameter (mm)	1.16 (1.07–1.26)	0.002	1.13 (1.02–1.25)	0.024
**Intraoperative variables**				
Total crystalloids infused (mL)	1.001 (1.001–1.002)	<0.001	1.001 (1.000–1.002)	0.031
5% albumin infusion	(0–inf)	0.981		
RBC transfusion	2.12 (1.26–3.46)	0.014	1.22 (0.61–2.47)	0.574
Urine output (mL)	1.001 (1.000–1.001)	0.004	1.000 (1.000–1.001)	0.662
**Echocardiographic parameters**				
Valvular disease	0.43 (0.04–1.75)	0.419		
E/A ratio	0.28 (0.10–0.74)	0.042	0.22 (0.05–0.95)	0.042
E/e’ ratio	1.13 (1.04–1.22)	0.013	1.16 (1.04–1.31)	0.011
TDI E velocity (cm/s)	0.84 (0.72–0.97)	0.054	0.99 (0.85–1.15)	0.889
Wall motion index	7.60 (1.56–51.77)	0.039	6.44 (0.64–64.83)	0.114

Prolonged hospital stay is defined as hospitalization of ≥8 d. RBC, red blood cell; CI, confidence interval; and TDI, tissue Doppler imaging.

## Data Availability

The datasets used and/or analyzed during the current study are available from the corresponding author upon reasonable request.
